# Requestioning the Indonesia Government's Public Policy Response to the COVID-19 Pandemic: Black Box Analysis for the Period of January–July 2020

**DOI:** 10.3389/fpubh.2021.612994

**Published:** 2021-05-10

**Authors:** Dumilah Ayuningtyas, Hayyan Ul Haq, Raden Roro Mega Utami, Sevina Susilia

**Affiliations:** ^1^Health Policy and Administration Department, Faculty of Public Health, Universitas Indonesia, Depok, Indonesia; ^2^Faculty of Law, Mataram University, Mataram, Indonesia; ^3^Biman Foundation, Depok, Indonesia

**Keywords:** black box analysis, COVID-19, government, Indonesia, health policy review, public health emergency

## Abstract

**Objectives:** Indonesia responded the COVID-19 pandemic slowly these last months. The recent reports shown that the rate performance of Indonesian government in handling COVID-19 posits at the 4th worst all over the world. Meanwhile, through responsive, strict, and strategic policy, some Asia countries pushed the elimination case by doing lockdown. This paper questioned how government respond this pandemic, tried to track down the unresponsive and slow decisions, and analyze them comprehensively trough policy system framework. Moreover, we also considered a few feasible and strategic recommendations to accelerate the pandemic responding.

**Methods:** To visualize the anatomy of problems in handling these pandemic responses, this work applied Easton's black box analysis in formulating and introducing public policy. The black box analysis would help us to portray and understood the interests, rationalities, and priorities behind introducing public policies which was implemented to handle this health problem. Besides, the policy triangle framework was used to analyze how environment influenced key actor in making decision.

**Results:** This analysis study discovered the conflict interests in formulating and implementing public policy in handling COVID-19. The public policies are negotiated, discussed, and formulated under black box that ignore transparency, and other good governance principles. Consequently, the substance of public policy represents a certain interest of policy makers, that may conflict with the others and often contradict to the constitutional-based public interests, that is public health. It was impacted the emergence of messy and uncoordinated institutions that implement the conflicted policies. Undeniably, this situation may spark counter-productive ways, attitudes, and actions of people in responding those ambiguous policies. Therefore, this work recommended revising the coherences norms and public policies; reforming the ministry of health in public health's paradigm context; and improving the integration and coordination of cross related institutions, creating a single data on public health, and changing a new paradigm of people, including improving collective awareness in responding and handling COVID-19 appropriately.

## Introduction

A new and threatening viral infection emerged in December 2019 and lead World Health Organization (WHO) to take serious steps to respond it. After some efforts, this global board assigned it as public health emergency (1). It also issued a public statement defining to be prepared by taking important measures to prevent the spread of the COVID-19 at local and national level. Through the Southeast Asian regional representatives, the WHO asked the countries to straight away to scale up all comprehensive efforts before the cases grow rapidly (1, 2).

Meanwhile, Indonesia government did not take the outbreak rapidly and seriously as evidenced by the dissemination of unclear and inconsistent information and unclear decisions. The Task Force Unit formed in March (3) and the first case appeared in the same month (4). Two months, amount of time passed by without a precise strategy to prevent the spread of COVID-19 in the country. Public could notice the government, attitudes, and statements that tended to minimize the scale of the problem and even asked them to take it easy. In the early time, the Government's warnings about the threat of news hoax were stronger compared to the alertness about the potential spread of the COVID-19.

Over 4 months since announcing its first case, the Indonesian government continually received sharp criticism for its inadequate efforts in dealing with the COVID-19 pandemic (5). The poor performance of the government was seen in some issues. One of them was the case numbers. The first cases are only two and the new case just appeared after 2 weeks later (4). Only in a month, the cases have been rising over 100 every month since last April. As of 31 July 2020, the official data confirmed 108,376 positive cases and 5,131 deaths (6, 7). Dealing with the outbreak surely needs a huge resource. However, out of the IDR 450 trillion budget prepared by the Central Government, the health sector only received IDR 75 trillion; as it turned out, most of the budget, as much as 150 trillion, was used for the recovery of the economic sector. All these raised questions about the Government's policy priorities and strengthened the opinion of some people that the establishment of the new normal at that time reflected the Government's tendency to save the country's economy first rather than focus on public health (8).

While the curve of COVID-19 cases continues to increase, the President of the Republic of Indonesia even announced that Indonesia would enter a new normal during which the country would have to learn to live with COVID-19, because the virus will not disappear (9). As a follow up on these directives, the Government then issued a new normal protocol (new normal) for offices and industries. This is considered an unfortunate development by some people, because the number of COVID-19 cases has not yet started to show a decline, and the determination of the so-called “new normal” is not entirely based on regulations from the WHO (10). Observations regarding the lack of integration and coordination as a measure of unpreparedness or government stuttering emerged not only in terms of data transparency, but also in many other things, including the ways by which several government institutions conveyed the risks through ongoing public communication. For example, it was about an Anti-virus necklace created by the Ministry of Agriculture and so on (11).

Regarding the current dynamics and facts showed the failures in handling the COVID-19 pandemic, it was the public policy that have power to control since early time. Public policy is a series of action taken or not taken by a government to solve public problems for the realization of public interest or to help people affected by the policy (12). Hence, the failure of public policies, including health policies, often becomes the basis for questioning the quality of such policies (13). National public policy setting takes place in the complexity of the process as a system, policymaking process as a system) (14, 15). Although intensively observed and analyzed, Easton (16) stated it still unclear how this governmental transformation process takes place in detail, and called this unclarity transaction process as *the governmental black box* (14, 15). Therefore, the purpose of this paper is to conduct policy analysis to answer and explain the following questions: (i) Is there stuttering and unpreparedness on the part of the Indonesian government in terms of taking action to prevent the spread of the COVID-19 pandemic in Indonesia based on the black box of policymaking process framework? and (ii) What recommendations can be given in the future for a better Indonesia?

## Materials and Methods

This study conducted by qualitative-interpretative approach. This method opened opportunities in using one or more ways for gathering, accessing or generating data: observing, with whatever degree of participating, interview in conversational mode; and the close reading of topic-relevant documents (17, 18).

We implemented a purposeful content analysis of available policy documents, programs, action plans, reports, press release, news related to COVID-19 in Indonesia, and from WHO. To select the valid data, documents and reports, we carefully picked from official website, for example: covid.go.id, kemkes.go.id, www.who.int, and many more credible ones. We also intentionally observed news with topics related to the study hypothesis with attention to objectivity. Therefore, we looked after information from national-scale media websites which release reports or news with the credible resource person, such as the President; the government representatives either from ministry, The Task Force Unit for COVID-19; or non-government coalition at the national level.

Regarding to the object of this scientific work, this is an analysis of public policy to gain alternatives and various implications for society. A public policy expert, William Dunn, wrote a definition of policy analysis as an applied social science discipline with various approaches to scientific methods and arguments to generate and transfer information relevant to policy so that it can be used as an effort to solve policy problems (13). Policy analysis is the activity of creating knowledge about and in the policymaking process, which includes researching the causes, effects, and performance of public policies and programs. The results of the analysis are then presented to public policy makers, who can use them to improve policy processes and performance (13).

### The Conceptual Framework

This study used the black box design and health policy triangle as the conceptual framework to understand health policy system constructed the pandemic management and how key actors made decision.

### Black Box-Based Public Policy in Handling the COVID-19 Pandemic

It is interesting to analyze public policies issued by the Indonesian government using the black box framework. This is because, like the black box in an airplane, which stores conversations in the cockpit and is used to reveal many secrets and their complexities in the aftermath of an airplane crash, the black box analysis can reveal the untold stories about the interrelations and dynamic interactions among elites and/or actors involved in the policymaking process.

**Figure 1:**
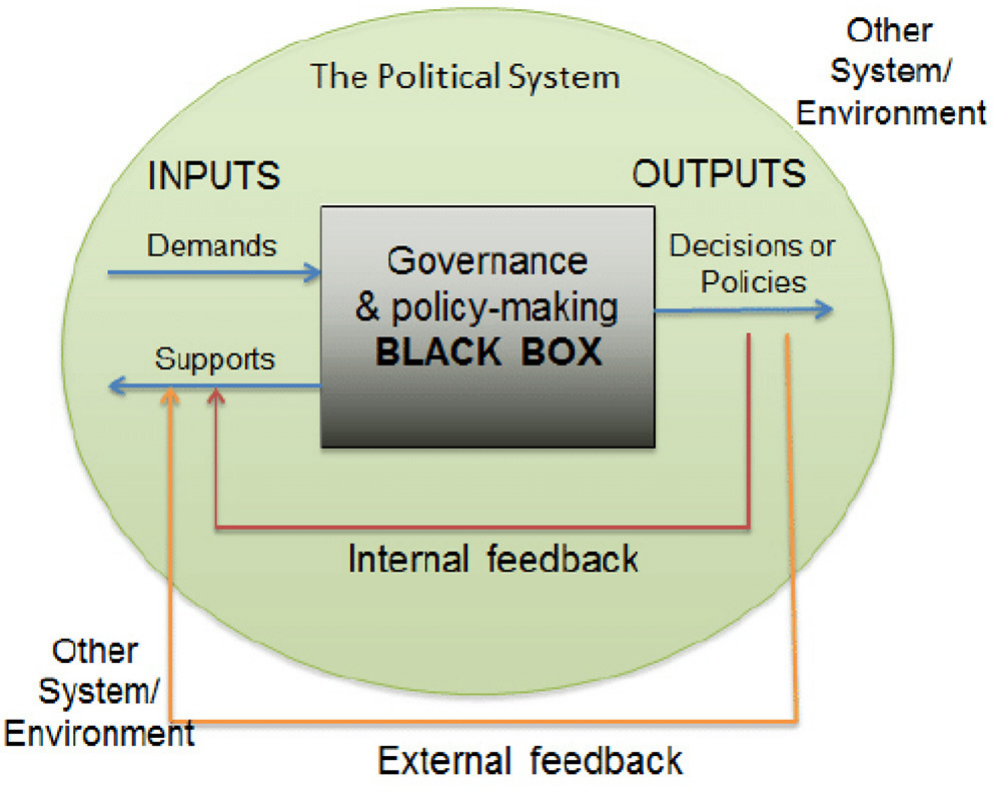
Analysis of Political System (David Easton, *A Systems Analysis of Political Life*, 1965). Source: researchgate.com.

Demands to the government and support from political parties and citizens become the inputs to the political system, as can be seen in Figure 1 (13, 14). Then, based on these inputs, the process of giving responses or making policies (policymaking) is initiated in the political system, with outputs that give birth to decisions and policies. This point is known as a black box, a stage wherein the interaction process takes place between the demands and support of various actors involved to produce a policy output. Within a system, the output itself can later provide feedback and become an input for further policymaking. The output created by following a certain demand will give birth to new support for the system itself. However, if the policy outputs generated do not match, then the system's subsequent instability can open opportunities for policy revisions (13).

The framework of systems analysis in Easton's political science discussed above is in line with the cybernetics theory developed by Talcott Parson in his study of legal sociology. Also known as structural–functional theory, it posits the existence of four sub-systems in shared life, namely, cultural, social, political, and the economy, as interrelated components that influence one another. All components of this sub-system have specific roles and functions that influence one another, especially during the formulation of public policies. In more detail, it can be explained that culture has the weakest determinative energy in constructing economic, political, and social patterns despite the fact that it has the most information in shaping social, political, and economic patterns (19–21).

In several theoretical and practical political studies, it is often the case that certain groups—especially the political elites—try to influence public policies to make them more profitable for their party. Such efforts can be seen from their maneuverings and games of power (power exercise), including their behaviors in grabbing or pursuing power (power-seeking) (22). Given that the political process focuses on accommodating and allocating values, it does require a balanced exchange or reconciliation between social components. A reconciliation of the differences of interests and requests (demands) is required to achieve or maintain the stability and cohesiveness in a bargaining position, to determine the value allocation authority, and to identify the reason for the allocation, which occurred in a process known as the black box of policymaking (23).

## Result and Discussion

### Assessment of Policy Alternatives and Their Implications

#### Aspects of Transparency and Responsiveness

**Figure 2:**
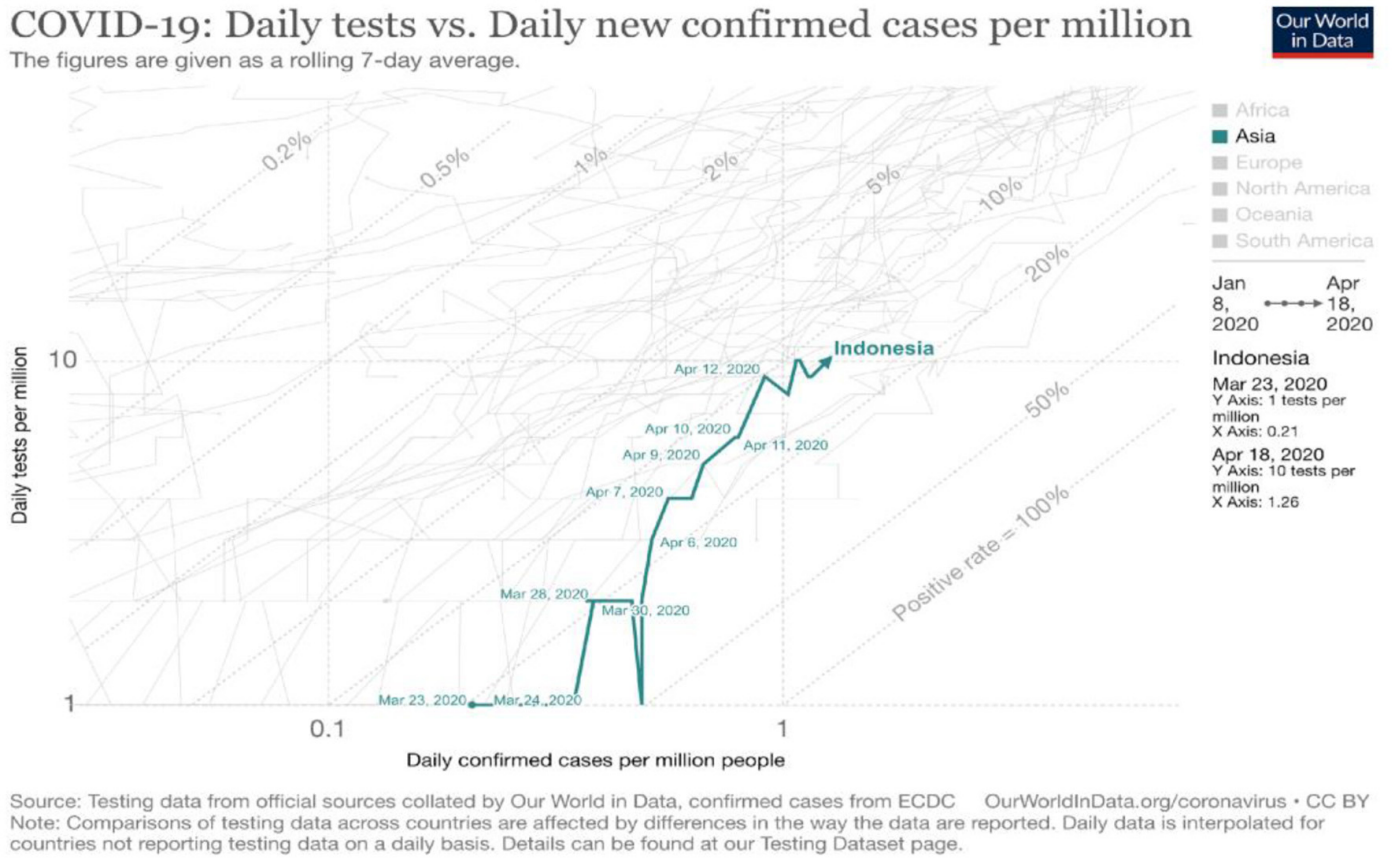
COVID-19 daily tests vs. daily new confirmed cases [Source: (26), Oxford University 2020].

Indonesia is thought to be the country with the worst COVID-19 testing performance in the world after Bangladesh (24). Within 1 month of announcing its first case in early April 2020, Indonesia had only carried out 14,354 or 52 per 1 million population tests, far behind other countries in the same period (25). Based on data obtained in April 2020, Indonesia has only conducted examinations on 10 people per one million population (26) (Figure 2).

The WHO, through a release issued on 6 July 2020, also issued its criticism (27). The WHO stated that priority examinations with the gold standard polymerase chain reaction (PCR) should be conducted to detect new cases, namely, Patients Under Supervision (PDP) and Person Under Monitoring (ODP), rather than to confirm the follow-up examinations of patients before being declared cured and discharged. The WHO also found that the death rates of PDP and ODP patients were surprisingly higher than the death rates of confirmed cases of COVID-19 (26). The impact of the country's dismal performance and capacity when it comes to COVID testing has made Indonesia one of the countries with the highest death rate for health workers in the world, with a death rate of 2.4% of the total 89 health worker deaths due to COVID-19 as of 13 July 2020. Indonesia is only trailing behind two countries, namely, Russia (4.7%) and Mexico (2.8%). As of August 1, several health professional organizations in Indonesia recorded that the number of health worker deaths due to COVID-19 had increased to a total of 153 cases (28).

About the Indonesian government's stuttering in handling the pandemic was allegedly due to the uncomplete, a unintegrated of the data. Surprisingly, concerning data transparency, the President admitted that the Government had deliberately not disclosed all data in the early months of the pandemic because it did not want to cause public anxiety and panic (29). The Government's non-transparent attitude has sparked the concern of many parties, including a non-government coalition (30). The representative person even said the way the government provides this information is far from fulfilling the constitutional rights of society. The transparency is an important turning point to create public trust and willingness to be involved and actively participated in dealing with this pandemic. However, it has been only a month since the previous announcement, The President through the Government Spokesperson for COVID-19 conveyed to the central and regional governments to be transparent to each other. The President further asked all relevant stakeholders to carry out effective, detailed, and transparent communication to all parties. The disclosure of the data should also be eventually integrated into the Acceleration Handling Task Force COVID-19 (31).

The difficulty in ensuring data transparency has significant consequences. On June 24, the Chairperson of the Expert Team for the Task Force for the Acceleration of Handling COVID-19 conveyed the results of Indonesia's work against COVID-19 for the past 3 months. The organization demonstrated a system called *Bersatu Lawan Covid* (BLC) or United Against Covid, which collected various data on handling the COVID-19 outbreak from the Ministry of Health (MOH), including data from Hospitals Online. Research Agency Network Laboratories and the Health Development, as well as the Public Health Emergency Operating Center under MOH. Data from BLC showed that the number of patients who died reached 11,477 people (51.5 people per 1 million population) (32). On the same day, however, the government spokesperson for handling COVID-19 reported on a live broadcast that the death rate was 2,500 people (14 people per 1 million population), placing Indonesia as the country with the highest death rate in Southeast Asia (32). The difference in the official data announced by the Government is ironic.

This is because even before the COVID-19 pandemic occurred in Indonesia, the *Satu Data Indonesia* (SDI) program—a programs that integrate data from multiple sectors as implementation of Presidential Regulation (PERPRES) Number 39 of 2019—was already launched to regulate the synergy between ministries/ agencies, central and regional, as well as government and society in implementing development (33). Thus, in this case, even though the aim of this program is to improve data governance to produce accurate, up-to-date, integrated, and accountable data, it has failed to become the foundation for effective and targeted policymaking for COVID-19.

The government spokesman for COVID-19 efforts also repeatedly issued controversial statements, thereby indicating the government's weak risk communication regarding the pandemic. One of the things that had caused a complicated controversy was the statement: “*Yang kaya melindungi yang miskin agar bisa hidup dengan wajar, dan yang miskin melindungi yang kaya agar tidak menularkan penyakitnya*” (The rich people protect the poor so they can live properly, the poor protect the rich so they do not spread the disease) (34). Following the absence of data on the number of ODP and PDP deaths, the spokesperson defended this by saying that the WHO did not ask for this data, so they did not feel the need to report. Sources from the Task Force said that even in the early days of the pandemic, the Task Force failed to obtain detailed data on the conditions of the outbreak, even though such data were very much needed to monitor the pandemic conditions and determine the next steps to be taken. Unfortunately, the slow integration of data has had complicated, far-reaching consequences for patient mapping, COVID-19 response, public trust, and most importantly, the possible protection of human lives (32). Several parties, including the London School of Hygiene and Tropical Medicine UK, have modeled estimates that there are far more positive cases of COVID-19 in Indonesia than that reported by the Government, which is only about 4.5% (35).

### Rationality of Prioritization

Yet, even as the curve of COVID-19 cases continues to increase, the President of the Republic of Indonesia has announced that the country will enter a new normal. The implementation of this new normal is regulated in the Decree of the Minister of Health Number HK.01.07/MENKES/328/2020 concerning the Guidelines for the Prevention and Control of COVID-19 in Office and Industrial Workplaces in Supporting Business Continuity in Pandemic Situations (36). The Ministry of Home Affairs also issued Decree Number 440-830 of 2020 concerning the Guidelines for the New Productive and Safe COVID-19 Normal Order for State Civil Servants within the Ministry of Home Affairs and Local Governments. This latest directive was issued even though the country has yet to meet some of the requirements delineated by the WHO for a country to create a new normal scenario, which include the following (10):

1. Evidence showing that COVID-19 can be controlled;
2. Capacity of health and public health systems, including hospitals, to identify, isolate, test, trace contacts, and quarantine;
3. Evidence that the risk of COVID-19 is minimized among vulnerable populations, especially in nursing homes, mental health facilities, and communities living in crowded areas;
4. Evidence that preventive measures are implemented in workplaces, including physical distancing, hand washing facilities, and other hygiene protocols;
5. Evidence that the risk of imported cases has been well-managed; and
6. Evidence that people have a voice and are involved in the new normal life.

Based on data from the WHO, until 8 July 2020, there was not one single province in Java Island, except for Banten Province, which had shown a drop in cases of at least 50% over the past 3 weeks since the peak of the last cases (27). The only province in Java that had reached the minimum detection rate was DKI Jakarta Province. This meant that the discourse and direction of easing policies to enter the era of a new normal were issued even though the emergence of new cases had not slowed down and the curve had not even sloped at all.

The accusation that the policy for handling COVID-19 was a “trade-off” between the interests of public health and the economy cannot be dismissed at the time of the determination of the new normal, which some parties believed was issued too quickly (37). The assumption that the Government is more concerned with the economy than the health sector is also associated with the view regarding the inadequate funding allocated for handling COVID-19 compared to other countries. The determination of the new normal under inappropriate situations and conditions has led some people to think that the Government is more concerned with the economy than the health sector. Furthermore, the budget allocation in our national expenditure budget is more focused on saving the economy by helping people whose incomes have been reduced or lost and by ensuring the availability of necessities. Indeed, public health in Indonesia, as a basic prerequisite for life, is not yet a priority for the Government. In fact, on March 31, the Government decided to increase the budget for financing pandemic response with a focus on economic recovery; from IDR 405.1 trillion, IDR 110 trillion was allotted for social protection, IDR 70.1 trillion for tax incentives and stimulus for People's Business Credit, and IDR 150 trillion for financing the national economic recovery program (37).

Some scholars have attempted to explain this phenomenon using the classical conceptual framework proposed by Wagstaff (38) regarding the poverty–health vicious cycle, which explains that poor health conditions affect the economic status of individuals through a loss of income and increased vulnerability to health care costs. Conversely, poverty causes health to also become worse, because the poor are vulnerable to suffering from various types of health problems (32). In relation to this, Indonesia's budget allocation strategy received criticisms, because it demonstrated that the Government was not focused on ensuring public health and safety against the threat of this pandemic. Despite the financial aid prepared for informal workers, such as day laborers, the benefits will be lost if they contract COVID-19 (33, 34).

### Inter-institutional Coordination and Integration

A few weeks after the term “new normal” was used in the context of loosening the PSBB to revive the sluggish economy, the Government then stated that such a term, which had been used so far, was incorrect. In fact, the term “new normal” is not sufficiently understood by the public. It makes the people focus only on the word “normal” when the actual situation is far from normal; worse, some even mistakenly interpret it as the return to old normal. Instead, the term should be interpreted as an adaptation of behaviors to the current situation. The behavioral adaptations in question include maintaining social distance, wearing masks, and washing hands frequently with soap, which are intended to limit or prevent further transmission. The public is asked to accept the fact that COVID-19 is still around us, but that people should feel that they are no longer at risk.

The consideration of the condition of public understanding and acceptance of the term “new normal” may have driven the Government to no longer use the term, and replace it instead with “adaptation to new habits.” The issuance of the policy on the use of this new term can be viewed positively, indicating the Government's willingness to make policy improvements. However, the rectification of this term also makes it appear that the Government “recognizes” its unpreparedness in various matters, including the determination of previous policies (35).

The issue of coordination and integration between agencies and ministries was retested in another incident. The curve of the number of cases began to increase quite sharply in relation to the observance of religious holidays in Indonesia. Indonesian people have a strong homecoming tradition during the Eid al-Fitr holiday, and they observed this even amidst the pandemic. At first, the Government had encouraged the people not to go home or return to their hometowns for this year's Eid celebration, mainly through a song sung by state officials entitled *Ojo Mudik*. This is important, because the mobilization of the majority of the population in the capital can cause the spread of COVID-19 to remote areas. Families in their hometowns also faced a high risk of receiving migrants from the city.

Unfortunately, the promotion carried out by these officials crumbled with a statement from the President in mid-April: “*Kalau pulang kampung boleh, mudik tidak boleh” (Back to home is permitted, homecoming is not)*” (36). This statement showed similar two terms that refer to situations where people will travel and increase the risk of transmission of COVID-19. It made confuse and difficult for many parties in the field because many people ended up deceiving the officers by taking advantage of the President's statement, whereas the Government had previously declared a ban on people from the regions to implement PSBB. Arguably, the confusion and ambiguity arising from this policy may have been responsible for the further increase in COVID-19 cases in Indonesia. This improper risk communication has led to the lax supervision of travelers from the capital. For example, on the Bandung–Tasikmalaya–Ciamis route, checkpoints for officers were visible, but at certain hours, there were no checks and even no officers. Thus, people who went home, carrying homecoming equipment on two-wheeled vehicles were free to drive in the area.

Another example that can be related to the Government's perceived stuttering in terms of the coordination between agencies and ministries during a pandemic is the continued implementation of the UTBK (Computer-Based Written Examination) last July. The UTBK was even held in areas with very high COVID-19 cases, including Surabaya. Thankfully, several local governments, such as that in South Tangerang, Banten Province, decided to postpone the UTBK, because they were still implementing PSBB. Of course, local governments that continued to hold UTBK exerted efforts to implement health protocols. The Surabaya local government, for example, implemented a free rapid test policy for students holding KIP as a step toward preparing for the UTBK implementation (10). However, criticisms regarding its effectiveness have been raised, because rapid tests are considered an inadequate measure to assess whether a person is truly safe from the COVID-19 infection. This situation reflects the perspectives of responsiveness, priority setting, and integration among government agencies, including the Ministry of Education and the Ministry of Health, as indicators of whether or not the Government is ready to deal with the COVID-19 pandemic in Indonesia.

### Structure-Function and the Leadership Role of the Ministry of Health

Determining the Status of Public Health Emergencies through Presidential Decree No. 11 of 2020, which concerns the Determination of Public Health Emergencies related to COVID-19, is a long-awaited regulation and is a relief for many parties. Nevertheless, it still raised criticisms because it only appeared several months after the WHO declared a public health emergency in January. Unfortunately, news emerged in the mass media that between these periods, several statements from the Government were considered counterproductive to the pandemic response, such as statements that you do not need to worry too much and just enjoy facing a pandemic and that there is no need to wear a mask if you do not get sick. More dangerous than the COVID-19 itself were statements that this was just a news hoax, which unfortunately came from institutional leaders and national level public officials closely related to the health sector. Needless to say, such statements affected the public's sense of crisis and urgency regarding the imminent dangers brought on by the transmission of the COVID-19.

Public assessment of the slow pace of government action, among others, also began with the implementation of Government Regulation No. 21 of 2020 concerning the Large-Scale Social Restrictions (PSBB) in the Context of Accelerating Handling of COVID-19, whose derivative regulations were stipulated in Permenkes No. 9 of 2020. This regulation positions the Ministry of Health as a party that will approve the submission of the local government for the determination of the PSBB status. However, different regional capacities in fulfilling various requirements and the formal administrative process for submitting PSBB may cause delays in its application, which is unfortunate because the situation is an urgent one. The PSBB is expected to reduce the curve of COVID-19 cases, but the reality on the ground does not work that way. For example, even before the policy of the central government related to PSBB was implemented, some provinces, including Papua Province, first initiated a lockdown by closing all access in and out of the province, in response to the provincial government's call to protect its citizens.

The focus of the policy strategy on public health resilience is not yet strong in terms of the function, structure, and leadership role of the Ministry of Health. That the responsibility of initial efforts to prevent, detect, and respond falls on the regional governments indicates the lack of robustness of the efforts implemented by the Ministry of Health. Delays in handling from the start can be a reflection of the less than optimal function, structure, and role of the Ministry of Health in terms of public health resilience. In fact, in a public health emergency, the public health paradigm (public health law) should be the commander who leads the issuance of various policies, strategies, and programs for overcoming COVID-19.

The PSBB is one of the policies issued in response to public health emergencies. Efforts to tackle the COVID-19 pandemic aim to ensure that Indonesia can develop strong capabilities in preventing, detecting, and responding to various threats to global health security. Therefore, in line with the GHSA agenda, the Indonesian government needs to place public health resilience (part of national security) as a top priority in every policy implementation in order to effectively respond to the COVID-19 pandemic.

Therefore, it is very unfortunate that in this situation of public health emergencies, there remains a continued scarcity of tools, facilities, and infrastructure for handling COVID-19, and even the active participation of various levels of society to collect donations to buy supplies cannot help augmenting these shortages. For instance, the shortage of personal protective equipment (PPE), which are urgently needed by medical personnel, is exacerbated by the high prices of supplies (e.g., masks, alcohol solutions, hand sanitizers, etc.) and essential PPE, such as hazard material suits, the price of which has increased by 20 times due to hoarding by speculators and opportunists, revealing the existence of a medical equipment mafia. The Government's weak response in ensuring the availability of much needed logistics (26) reflects the capacity and effectiveness of the Ministry of Health's leadership, structure, and function, which are greatly tested in this context.

Various policy choices and their implications, according to the framework of the policymaking process remind us of the political behaviors of elites/policymakers as an important factor influencing policy output (e.g., explaining why policies frequently change or are revised). Departing from this concept, we use a system approach to analyze these elites/policymakers as a component of policies for handling the COVID-19 pandemic in Indonesia.

The Table 1 shows the process of determining government policies to face a pandemic, according to Kivits. It explains the dynamics of stakeholders and their respective positions in the framework of power and urgency. The discussion of stakeholders and their relations to the influence of power and urgency is a very dynamic concept that can change rapidly as long as the policy lasts (37). For example, when stakeholders are exposed to coercion, whether under normal conditions or not, that power can be sought but can also disappear.

**Table 1:** Analysis of Easton's system component mapping (input-process-output-environment).

**Elite/policy actor**	**Positioning/expected roles of actors**	**Positioning/roles of actors in reality**	**Support/demand/ resources**
WHO	• Direct and coordinate local and international authorities in health efforts • Determine standards and guidelines • Coordinate international responses to public health emergencies • Assist the national government in improving health services • Cooperate with other specialized agencies if necessary • Provide health assistance to countries in need • Encourage and assist the implementation of research	• Diplomacy and dissemination of the latest accurate information related to the pandemic is suspected of being biased against China • Determining COVID-19 to be PHEIC is a bit late than it should be • Ensure that vital supplies reach health workers • Conduct training and mobilization of health workers through the platform Open WHO Leadership • Identify research priorities in the search for vaccines	Authority
Community/Religious Leaders	• Help communities avoid hoaxes and misinformation • Help convey health messages • Help remove the stigma focused on a group of people • Able to shift community actions in the desired direction • Become a pattern for the community to behave in the “right” life, including in terms of healthy living behavior during the pandemic	• Keep holding the *Ijtima Ulama Dunia* in Gowa, South Sulawesi, during the pandemic • Issue fatwas related to the implementation of worship during the pandemic • Allow Eid and Eid al-Adha prayers in houses of worship/fields in the COVID-19 (Islam) free zone, and appeals to carry out worship from home (non-Islamic) • Raise funds for victims affected by the pandemic • Issue guidelines/protocols of worship in houses of worship during the *new normal* era	Community trust
NGOs	• Fight for community aspirations • Social control of problems • Raising social and environmental issues	• Government advocacy to improve response to the pandemic • Establish a task force for handling COVID-19 within their respective organizations • Utilize organizational resources to help communities in need • Provide education and dissemination of important and accurate information related to the pandemic • *Crowdfunding* to help fulfill various funding needs (e.g., PPE) • Develop research related to pandemics	Organizations Social control
Higher Education/Research Institutes/Academics	• Input on government policy directions • Socialization and public education • Conduct pandemic-related research	• Conduct webinars, trainings, and scientific discussions to help solve pandemic-related issues • Conduct various research (e.g., medical devices, vaccines, drugs, etc.) to help deal with pandemic-related issues • Engage as a *key stakeholder* for the government to determine the direction of policies, strategies, and programs for handling the pandemic	Human resources Policy brief
Government (President)	• Main regulator • Leaders and commanders/navigation related to pandemic response • Collaborate with other countries in resolving pandemics • Involve pentahelix in resolving pandemics	• Establish a task force to handle the pandemic • Approve bureaucratic flow, use of inappropriate terms, and delays in handling pandemics • Direct every state institution to rush efforts to deal with the pandemic	Authority Legislative Regulation
Dewan Perwakilan Rakyat (DPR)/Legislative Board	• Budgeting • Supervision	• Ratify bills that are not a priority for handling the COVID-19 pandemic • Do not carry out comprehensive supervision related to handling pandemics	Role and the authority of the policy making
Ministry of Health	Coordinators, regulators, and implementers (to a certain extent) related to the health aspects of the pandemic response	Delay in announcing pandemics • Notes on limitations and inaction in response to the pandemic in the early stage • Criticism of the principle of efficiency and effectiveness of M&E (monitoring and evaluation) in handling the pandemic	Authority Regulatory
Badan Nasional Penanganan Bencana (BNPB) National Board for Disaster Management	• Implementing core government policies trough integrating other many parties roles and data sources during the pandemic	• Providing guidance and direction for disaster management efforts covering disaster prevention, disaster emergency response, rehabilitation and reconstruction in a fair and equal manner.	Authority Regulatory
Ministries other institutions	• Support core policies from the government (president)		Authority Regulatory
Entrepreneurs	• Investment • CSR • Anticipation against possible policies that can affect the companies' economic sustainability	• Changing the pattern of business continuity during the pandemic • Helping people/employees who are affected by the pandemic • Carry out community service to help those affected by the pandemic	Capital Companies Manpower
Local Government	• Implementing policies at the provincial/district/city levels	• Stuttering implementation of top-down policies of the central government • Take initiative in handling pandemic-related issues • Conduct innovations that often do not have a good impact on the pandemic	Authority in the regional Regulations in region # Adjusting the central direction

The first factor in Mitchell et al.'s (39) model is power: when a stakeholder has access to coercive, utilitarian, or normative means of power, it can impose its principles onto the relationship. Access to power, or the means to exert power, is often variable and is not in a steady-state (40). Power may be gained, as well as lost, over time. Within the stakeholder relationship, it is therefore important to be aware of the power relationships between stakeholders and how these relationships might change over time (34). This also determines the degree of importance attached by stakeholders to certain issues; thus, the degree of importance varies for each stakeholder.

The description of elite interaction in the determination of policies for handling the COVID-19 pandemic, as described in the Table 1 shows the birth of public policies that take place as a system, with several internal and external factors influencing its determination. Models that identify several factors that influence the political behavior of individual political elites can be visualized in the following combinations of approaches: (i) indirect socio-political environment, such as political systems, economic systems, cultural systems, and mass media; (ii) the direct socio-political environment that influences and shapes elite personalities, such as family, religion, school, and social groups; (iii) personality structure, which is reflected in individual attitudes; and (iv) direct socio-political environment factors in the form of situations, namely, conditions that directly affect actors when they want to carry out an activity (41). The roles and positions of the WHO, Community/Religious Leaders, NGOs, Academics, the President, DPR, BNPB, Entrepreneurs, and Local Governments as actors or elites in determining policies to handle the COVID-19 pandemic are directly or indirectly influenced by environmental and structural factors.

Therefore, realizing how dynamic power relations take place is very important in mapping out the socio-economic power in the policymaking environment; in this way, the outputs of policies take into account the support resources and the demand they have. Furthermore, the policymaking process should take place with an awareness of the phenomenon of the black box of policymaking; it should not only be dominated by the narrow interests of political elites who bargain for positions and interests. For example, economic interactions between the government and the business sector can be seen in the tourism incentive policies issued during the pandemic. This policy, which is diametrical, contradicts the rationality of handling COVID-19. In other words, while other countries in the world are struggling to mitigate the effects of COVID-19 pandemic, the Indonesian government has allocated a budget of up to IDR 72 billion for foreign influencers to promote tourism and to increase flight escalation and interactions. It cannot be denied that this government policy shows the dominance of economic considerations without considering the country's ability to face a pandemic (33). Snippets of the main policies issued by the Government in dealing with the COVID-19 outbreak in the following table can also be understood using the black box of policymaking approach.

The Government must optimize the function and role of the Ministry of Health in preventing, detecting, and responding to the disease. In handling this pandemic, the Government has positioned the National Disaster Management Agency (BNPB) in the front line, in collaboration with the Ministry of Health and with support from other ministries and state agencies. Institutionally, the Ministry of Health's task is not only to issue a PSBB policy but also to prepare and formulate health policies so that the pandemic will end quickly (42). Normatively, the analysis of government policies in overcoming the COVID-19 pandemic in Indonesia can be reviewed in relation to the global standard regulation, namely, the IHR 2005 issued by WHO and ratified by Indonesia as a mandate to be implemented. The global agreement in the prevention of transnational diseases requires that each country must have adequate capacity, both in routine conditions and during public health emergencies, at ports, airports, and state land border crossings (PLBDN), especially in conditions that are designated as a public health emergency of international concern (PHEIC). The WHO has prepared assistance in the form of cooperation between countries in the evaluation, assessment, and capacity building of public health. Such an assistance also includes supporting countries in identifying sources of funds needed to develop and maintain the country's capacity. The enactment of this IHR (43) is typically followed by guidelines, instructions, and procedures to carry out routine inspections at ports, airports, and land borders (42). This 2005 IHR Agreement should be the basis of reference for the Indonesian government in formulating public policies in response to the spread of COVID-19.

At the national level, Indonesia is also bound by the Health Quarantine Act. Even before that, Indonesia has implemented Law No. 4 of 1984 concerning plagues; Law No. 24 of 2007 concerning Non-Natural Disasters, Epidemics, and Disease Outbreaks; and Law No. 23 of 2014 concerning the Sharing of Concurrent Affairs in the Health and Disaster Sector. Unfortunately, when the COVID-19 pandemic hit the world, Indonesia did not proportionally appreciate the various normative provisions above. Even at the level of implementation, Indonesia was considered inconsistent and stuttering in responding, formulating steps, or taking action to overcome the COVID-19 pandemic. This can be seen from the many regulations issued by the Government that complicated the handling of the pandemic, which caused uncertainty, instability, and confusion not only at the conceptual and administrative level but also at the level of implementation.

The analysis of various policy products as well as the roles and respective policy actors listed in the Tables 1, 2 constitutes a policy review with a triangle of policy framework (content, process, and actors). However, the various complexities of the above problems can be deepened by using legal system theory introduced by Lawrence M. Friedman. This theory holds that the effectiveness of legal safeguards rests on at least three components of the legal system, namely, (i) legal substance, (ii) legal structure, (iii) legal culture. The three components in the legal system are important prerequisites for implementing all public policies (44). These three components must be interrelated and interact in a coherent manner. Otherwise, the incoherence among these three components can have negative implications and can lead to counterproductive government policies.

**Table 2:** Main government policies in facing the COVID-19 pandemic.

**No**.	**Policy**	**Date in charge**	**Title**	**Responsible**
1	Presidential Decree No. 7 of 2020	13 March 2020	Presidential Decree (KEPPRES) on the Task Force for the Acceleration of Handling of COVID-19	Task Force for the Acceleration of Handling COVID-19
2	Presidential Decree No. 9 of 2020	20 March 2020	Presidential Decree (KEPPRES) concerning the Amendments to Presidential Decree No. 7 of 2020 concerning the Task Force for the Acceleration of Handling of COVID-19	Task Force for the Acceleration of Handling COVID-19, Synergy between Ministries and Institutions
3	Presidential Decree No. 11 of 2020	31 March 2020	Presidential Decree concerning the Determination of Public Health Emergency COVID-19	Task Force for the Acceleration of Handling COVID-19,
4	Government Regulation No. 21 of 2020	31 March 2020	Government Regulation (PP) regarding the Large-Scale Social Restrictions in the Context of Accelerating the Handling of COVID-19	Ministry of Health
5	Presidential Decree (KEPPRES) concerning the Determination of Non-Natural Disaster for COVID-19 as a National Disaster	13 April 2020		Task Force for the Acceleration of COVID-19 Response
6	Permenkes No. 9 of 2020	3 April 2020	PSBB Guidelines for Handling COVID-19	Ministry, Local Government
7	SE Minister of Religion No. 6 of 2020	6 April 2020	Guidelines for Ramadan and Eid Al-Fitr 1 Syawal 1441 H in the Middle of the COVID-19 Pandemic	Ministry of Religion, Society
8	Kepmenkes No. HK.01.07/Menkes/382/2020 Year 2020	19 June 2020	Health protocol for People in Public Places and Facilities in the Context of the Prevention and Control of COVID-19	Ministry, Society
9	Joint Decree of the Minister of Education and Culture and Menparekraf No. 02/KB/2020 and No. KB/1/UM.04.00/MK/2020	02 July 2020	Technical Guidelines for the Prevention and Control of COVID-19 in the field of culture and creative economy during the determination of public health emergencies related to COVID-19	Stakeholders of education and tourism/creative economy
10	Law No. 2 of 2020	21 May 2020	The Stipulation of Government Regulation in Lieu of Law Number 1 of 2020 concerning the State Financial Policy and Financial System Stability for Handling the COVID-19 Pandemic and/or in the context of dealing with threats that endanger the national economy and/or financial system stability into Law -The Act	All components related to the financing during COVID-19 pandemic

The large number of complex problems found in these three levels further reflects the ineffectiveness of policies for handling COVID-19 in Indonesia. An example of a problem at the structural level is the lack of synergy or coordination among institutions implementing the policies for handling COVID-19. The policies that have been made by the Government seem to be fading, for various reasons, so that even the officials at the scene of the incident are unable to take firm action against the people who violate the rules. This situation also reflects the weakness of law enforcement agencies and prevailing policies. Therefore, the Government's weak monitoring and evaluation of policy implementation indicates its inability to formulate a comprehensive and coherent legal substance.

Certainly, there are positive policies issued by the Ministry of Health that should be appreciated. For example, it initiated to increase ASEAN health sector cooperation in handling COVID-19 (45). This meeting resulted in the successful adoption of the Joint Statement of the ASEAN Health Ministers in increasing the collective response to COVID-19 in the ASEAN region. The Joint Statement outlines a commitment to continue to exchange data and information on the development of COVID-19 through established cooperation mechanisms, to coordinate contact tracing and case investigations through bilateral and regional mechanisms, and to share technical materials and mobilize resources in supporting national and regional health systems (45). However, in handling the COVID-19 pandemic, the BNPB appears as a “war commander,” thus leaving the Ministry of Health far behind.

Another criticism is that there is a need to strengthen the focus of empowering public health as a basic value. It is fitting for the public health paradigm or the public health law to become the commander in charge of the issuance of various policies, strategies, and programs to overcome COVID-19, in line with the Presidential Decree/Perpres 81 concerning Public Health Emergencies. The Ministry of Health must play a major role in ensuring health resilience in Indonesia, strengthening the country's ability to prevent, detect, and respond according to the IHR 2005.

The criticisms that should be addressed not only to the government but to overall governance have to do with the questions of how the efforts to disseminate the public health paradigm in the mainstream have been carried out all this time, whether there has been a systematic process from upstream to downstream (including educational institutions), and whether there are any legal aspects and regulations that form the basis of public health policies to strengthen health resilience as part of national resilience, especially considering that the pandemic is a global threat and public readiness to face it is determined by health resilience. However, the development and strengthening of national health and resilience is not a short project but a long journey.

In order to support the Ministry of Finance deal with COVID-19, in March 2020, the Government prepared a budget of IDR 75 trillion for the health sector (44). This budget is part of the IDR 405.1 trillion stipulated in the government regulations in lieu of laws on financial policies and financial system stability. The funds will be used for protecting the health workers; securing adequate supply of PPEs; testing; purchasing of reagents, ventilators, and other needs; and providing incentives for frontline health workers. However, until the end of June 2020, only 1.53% of the budget had been disbursed due to the constraints in the verification process and the slow bureaucratic flow, resulting in several health workers complaining about not receiving the incentives promised by the government. The disbursement of this budget also has a high potential for corruption, because its use and monitoring have not been implemented in a regular and transparent manner. In fact, the implementation of state financial management must be based on the principles of good governance, *one* of which is the element of transparency.

The priority setting aspect in policymaking should also be considered, because there are various factors that can change according to political conditions and pressures applied during the policymaking process. With this approach, the success achieved in determining policy priorities as well as the process and structure of decision-making will be more profound. In this context, we need a more evidence-based policy to respond to existing policy failures or success. Even though, many countries are not prepared to handle this sudden outbreak, referring to International Health Regulation State Party Annual Reporting (IHR SPAR) (44, 45). Hence, we still could learn from certain country which successes in preventing the massive spreading of the virus. For example, Vietnam that was rapidly prevent the outbreak by providing isolation place, integrating data; and engaging scientists and experts to prevent the viral spread and eliminate cases (46). In addition, similar situation in Indonesia might be seen in Italy where this pandemic turn into a disaster due to the highest number of death case. A scientific review mentioned that the profound obstacles were including how the leaders could not recognize the threat of this virus, then organized a systematic response to it (47). Another study's result showed a related situation that early reports on the spread of COVID-19 and adequate risk assessment can help inform government representative in effort to combat its progression. From the two countries, it could be lesson learn that primary strategies which are integrating resources rapidly; and leading transparent and accountable policy making processes are leadership capacity reflection in handling this pandemic.

Thus, in the future, it is hoped that the policies made will be a collaboration of conditions in the real field and science-based policymaking (48). The evidence-based public policy approach should be the basis in setting public health policy priorities, as it gives value to the importance of evidence (data and facts) in the formulation of public policies aside from opinions that are influenced by other interests, such as economy, politics, personal interests, power, and so on.

## Conclusion

The analysis conducted above helps prove the hypothesis of this study: The Indonesian government is not fully ready to face the COVID-19 pandemic. The determination of the status of public health emergencies is usually a strong message that the public health paradigm and approach are the “commanders” leading the war against the pandemic. This is explained by the fact that this pandemic is a reflection of the public health situation. Comprehensive and integrative handling should be emphasized, not only in health services but in other important aspects of behavior, environment (social, economic, political, cultural), and genetic factors (heredity) (48).

Apart from the diametrical policies above, the model and flow of communication and information as well as the system referral in the health service structure itself are not yet ready to face the COVID-19 pandemic. This can be seen from the processes and indicators in the health services, ranging from personal quarantine, hospital quarantine, and regional quarantine. To date, the PSBB's functions overlap with others, indicating the lack of adequate control, monitoring, and evaluation; thus, the implementation of PSBB is not optimal. The lesson that can be learned is the urgent need to review institutional structures so that they can develop a greater capacity for public health resilience. Enriching the Ministry of Health to become the Ministry of Public Health with systemic integration and a policy strategy approach should be seriously considered due to the urgent situation in the country.

Enforcement of public policies in handling COVID-19, whether in the form of the PSBB Law, government regulations regarding public health emergencies, or management of financial policies in handling COVID-19 and others, must not be contrary to the constitution. The ensuing public policies must not take away the fundamental rights of the people in maintaining the sustainability of daily life, making them a paradox of public policy. Therefore, the process of formulating public policies that are directly related to public health must be based on the principle of transparency, including the disclosure of accurate and correct facts, data, and information based on scientific reasons, public reason, public rationality or common sense.

The process of formulating and establishing government policies in dealing with the COVID-19 pandemic is taking place in a very dynamic and rapidly changing context. Many policy changes have not maximally considered the importance of public safety and health as top priorities. The influence of various interests, such as economy and politics, is still too dominant in the formulation and determination of government-led health policies. This situation can be explained by the delays, stuttering, and confusion among policy actors, officials and the communities (both at the central and regional levels) in anticipating, responding, addressing, and dealing with the spread of COVID-19, while the epicenter of COVID-19 continues to move to the regions. Institutional strengthening through the validity (scientific authority) and the credibility of public information related to the substance of handling COVID-19 determines the trust of all components of the nation and motivates everyone to move together.

Regardless evidence and theories supported. We are aware of the limitation of our study as analytical work. First, the idea was built at the beginning of the notification from WHO about a potential outbreak of a new disease. We expected that the government took a fast response through strategic decisions and technical instructions to prevent the entry of the new disease into Indonesia. Second, we conducted this study's hypothesis after seeing the government who took steps to deal with the new disease 2 months after the notification. This study could not capture the possibility of significances the changes and responses from the government, so the approval of the hypothesis would only be relevant for the time being. Then, this review was written in the first 4 months of handling the pandemic in Indonesia with the many social restriction regulation. As a result, we encountered limited data, other source information, and methods as the triangulation mechanism.

### Actionable Recommendations

The failure of policy development, starting from the formulation, implementation, and evaluation stages, accompanied by the lack of transparent and rational public policy principles as well as limited facilities, infrastructure, and human resource capacity in the field of public health, all have the potential to threaten public safety and health. Thus, several recommendations compiled by the author are as follows:

- The Ministry of Health must be reformed the paradigm with strong Public Health principle. The word “public” shows that parting with the community becomes more secure. This also avoids the Ministry of Health's sole focus on the medical aspect and enable it to strengthen health resilience as part of national resilience efforts, especially in terms of prevention, detection, and response. This is in line with the 2005 IHR issued by the WHO and also shows Indonesia's real participation as a member of the GHSA troika.
- Institutionally, the Government needs to re-examine and restructure the roles and functions of the Ministry of Health's institutions so that they play a more substantive role and function in handling COVID-19 with a systematic and integrative public health approach. This is an important step in increasing the credibility of public health resilience.
- The SDI program should continue to be the source of coordination and integration of the state, and assurance is the only key holder of health data within a clear and measurable line of command. This is expected to make data more adequate in preparing and strengthening infrastructure from all aspects in the field of public health, especially in responding to the COVID-19 pandemic.
- The health paradigm should be strengthened, because the WHO determined the quality of the Indonesian government's response to public health emergencies. This means educating public health advocates to become policy influencers and intensifying their involvement so that public awareness about public health rights in addition to obligations can be significantly increased.

## Author Contributions

DA conceived the manuscript idea with the support of HH, RU, and SS. HH wrote the introduction part. DA and SS wrote the methodology and analysis, meanwhile SS collecting and concluding the findings. SS prepared the figures. DA collected and integrated all drafts to become a full manuscript. All authors contributed to the article and approved the submitted version.

## Conflict of Interest

The authors declare that the research was conducted in the absence of any commercial or financial relationships that could be construed as a potential conflict of interest.
